# Far Ultraviolet Detector Standards

**DOI:** 10.6028/jres.092.011

**Published:** 1987-04-01

**Authors:** L. Randall Canfield, Nils Swanson

**Affiliations:** National Bureau of Standards Gaithersburg, MD 20899

**Keywords:** calibration, detectors, far ultraviolet, photodiodes, photoionization, quantum efficiency

## Abstract

A description is given of the NBS program in which special photodiodes for the far ultraviolet spectral region (5–254 nm) are made available as transfer standards. These detectors are calibrated in terms of quantum efficiency (photoelectrons per incident photon) as a function of wavelength. Descriptions are also given of the calibration principles, calibration systems, and photodiode types involved in this program. Calibrations reference to the photoionization of rare gases.

## 1. Introduction

During the period 1955–1965 there occurred a rapid increase in interest in scientific activity in the far ultraviolet, primarily due to two developments. First, advances in both vacuum and optical technology had led to the development and manufacture of convenient instruments (spectrographs, monochromators, etc.) with which many new experiments could be successfully conducted in the far ultraviolet. Second, the accomplishment of operating spacecraft had provided the opportunity to pursue solar and astronomical studies beyond the absorption of the earth’s atmosphere, hence into the far ultraviolet. With the great increase in far ultraviolet activity came the parallel need for radiometric capability in this region. The situation became rather obvious when it developed that experiments orbiting on different spacecraft, but observing the same phenomena, were recording vastly differing flux levels. Clearly a common, accurate radiometric base was needed and NBS undertook the establishment of a program which would lead to transfer standard detectors for the far ultraviolet.

The far ultraviolet detector radiometry program attempts to furnish transfer standards capable of determining absolute flux levels in the spectral range 5–254 nm. These standards should be relatively stable, simple to use, and within the typical laboratory budget. Calibrations furnished with these standards should be state-of-the-art in accuracy. Improvements in accuracy and stability should be constantly sought and incorporated when possible.

Two detector types (described in section 2) are now available from NBS as calibrated transfer standards covering the spectral regions 5–122 nm (probable errors 8–15%) and 116–254 nm (probable errors 6–10%). Users are furnished with the quantum efficiency of their detector as a function of wavelength (with quantum efficiency defined as the number of photoelectrons per incident photon).

Additionally, special detectors which do not lend themselves to convenient on-site cross-calibration may be calibrated at NBS if the detectors merit radiometric application and if the NBS facilities are suitable for the particular device.

It is hoped that eventually all far ultraviolet detector calibration activities can be conducted at the NBS Synchrotron Ultraviolet Radiation Facility (SURF-II), using its calculable flux as the radiometric base. This would eliminate the weakest link in the calibration chain, the thermopile, and might also lead to the elimination of the need for working standards. Even before this is achieved there are several areas in which improvements may be made. Materials studies may lead to windowless detectors with better spatial uniformity, temporal stability and improved quantum efficiencies. It is hoped that the short wavelength limit of calibrations can be extended and that the uncertainties of calibrations at all wavelengths can be reduced.

## 2. Description of Detector Types

### 2.1 The NBS Windowless Photodiode (5–122 nm)

The NBS windowless photodiode transfer standard detector is the result of temporal stability and spatial uniformity studies which were conducted at NBS in the 1960’s on several likely choices for a photocathode material suitable for use in an open vacuum photodiode [1].^1^ The spectral region of interest was primarily at wavelengths shorter than the transmission limit of magnesium fluoride, so as to extend the range covered by sealed photodiodes.

#### 2.1.1 Photocathode Material

The material of choice for the photocathode was and is aluminum with the natural oxide thickness artificially increased. Vacuum deposited aluminum (99.999% pure) samples on quartz substrates are anodized to increase the natural oxide thickness for use as photocathodes in NBS far UV windowless transfer standard photodiodes. The increased oxide (~15 nm thick) improves the stability of the photoyield over that of the deposited aluminum by preventing any further oxide development, and achieves more complete absorption of the incoming radiation, thus reducing wavelength-dependent variations in the photoyield caused by optical interference. (The aluminum has a very low coefficient of absorption in much of the spectral range and hence acts like a transparent film bounded on each surface by an absorbing film.) A description of the method used to accomplish this follows.

The anodizing method used has been described in the literature [2]. A pH 5.5 bath of tartaric acid is used with a 99.999% purity aluminum cathode. The bath is prepared by dissolving 3% (by weight) powdered tartaric acid in distilled water. The pH is measured and adjusted by the addition of small amounts of either extra tartaric acid or ammonium hydroxide, depending on whether more or less acidity is needed.

A simple teflon fixture holds the sample in contact with a pure aluminum wire at the edge of the circular substrate, so that most of the sample may be lowered into the bath without wetting the contact point. Electrical connection is made to this wire (+) and to the cathode wire (−) from a well-regulated power supply which has been preset to 10.5 V and current-limited to about 10 mA.

#### 2.1.2 Photodiode Assembly

**Figure 1:**
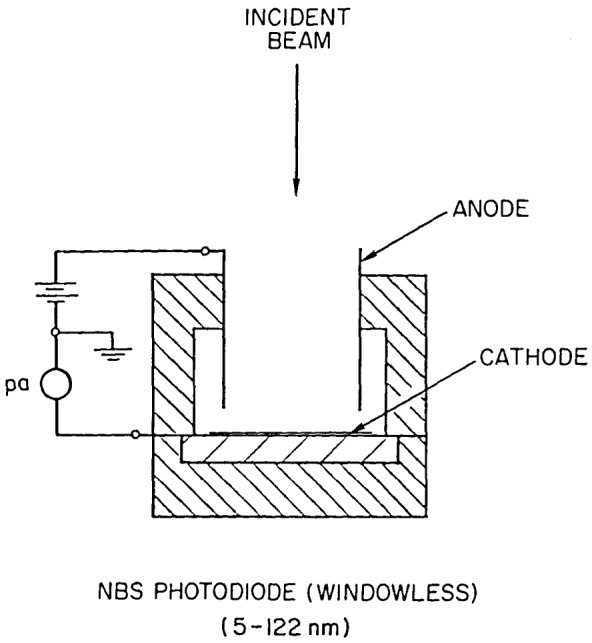
NBS windowless photodiode.

Figure 1 shows the configuration of this photodiode. A cylindrical anode is suspended very near the cathode, with a machined teflon component providing support for both. The photocathode is electrically connected to a machined piece of aluminum on the opposite side of the substrate by aluminum foil. Provision for physical location of the whole device is made by a threaded hole in the rear of the machined aluminum piece. This mounting is at cathode potential and must be well insulated from ground. The entire device is intended for use in vacuum.

#### 2.1.3 Operating Characteristics

**Figure 2:**
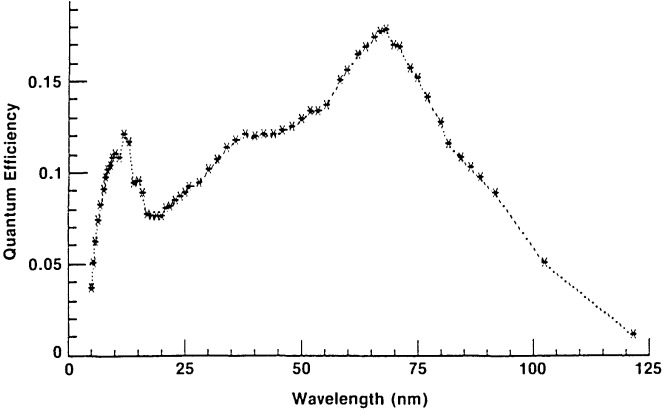
Typical quantum efficiency of NBS windowless photodiodes.

Incident far UV photons cause the photocathode to emit low- energy electrons, which are accelerated away by the electric field established by the anode potential of 60–100 V. The rate of emission is measured with a suitable calibrated picoammeter. The usable range of photocurrents is roughly from 10^−9^ to less than 10^−15^ amperes. (The “dark” current—mostly thermionic emission—is known to be less than 10^−15^ amperes, but has not been measured.) From a table of quantum efficiencies (electrons per incident photon) given in the NBS Report of Test which accompanies each photodiode, the flux rate at the surface of the photocathode may be derived. Typical efficiencies range from a few percent at 5 nm to a peak value of about 20 percent in the 60–70 nm region, then back down to about 1 percent at 122 nm (see fig. 2). The photocathode surface, being unprotected from outside contamination, may change in efficiency due to such exposure, so it is important that potential sources of contamination be recognized and controlled. One should also ensure that the photocathode is protected from charged particles during operation, since any such particles arriving at the photocathode would result in an incorrect assessment of the radiant flux.

### 2.2 Windowed Photodiode (116–254 nm)

During the history of the NBS far ultraviolet transfer standard detector program several suppliers of windowed detectors have been used. Initially satisfactory photodiodes were obtained from the Stanford Electronics Laboratory at Stanford University and from EMR Photoelectric in New Jersey,^2^ but in recent years both of these sources have ceased production of devices made to NBS specifications. We now obtain windowed photodiodes from the Electronic Vision & Systems Division (EVSD) of Science Applications International Corporation, which acquired the necessary competence as a result of a NASA contract to fabricate Digicon detectors for use in spacecraft.

#### 2.2.1 Physical Characteristics

**Figure 3:**
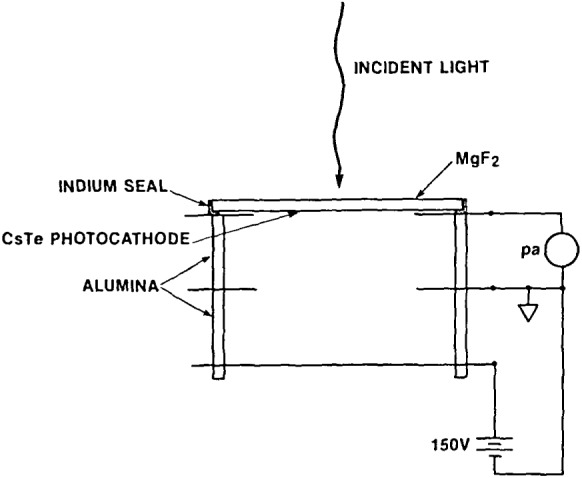
Schematic and wiring configuration of EVSD photodiode.

The photodiode supplied by EVSD (fig. 3) is known as their Model 54-0-000 and utilizes a semi-transparent cesium telluride photocathode deposited on the inner surface of a magnesium fluoride window. The device is fabricated in ultra-high vacuum with the photocathode being formed remote from the photodiode body, and the window/photocathode joined to the body by an indium alloy seal in the same vacuum. The body is made of OFHC copper electrodes brazed to ceramic spacers, with the center electrode isolating the cathode from anode leakage, and the rear electrode serving as the anode.

#### 2.2.2 Operating Characteristics

A supply of 150 V is attached to the anode and a calibrated picoammeter measures cathode photocurrent (fig. 3). The center electrode is normally grounded. Maximum photocurrent should be kept at less than 10^−8^ amperes and the magnesium fluoride window must be maintained free of contamination. Frequent cleaning of the window may also degrade its transmittance, and should be avoided as a routine measure.

### 2.3 Rare Gas Ionization Chamber

**Figure 4:**
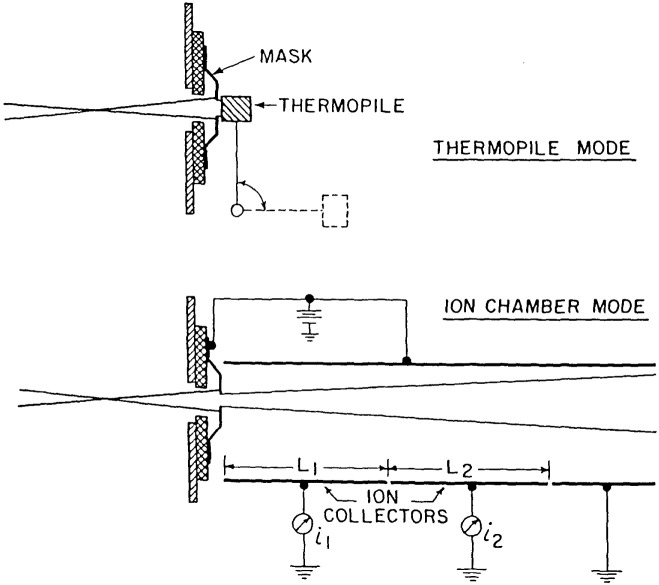
Configuration of thermopile and ionization chamber detectors.

The absolute detector which forms the basis for all present far ultraviolet detector calibrations at NBS is the rare gas ionization chamber. The basic operational principles and demonstration of the absolute nature of this detector have been discussed in the literature [3–5]. The design used at NBS (seen in fig. 4) has two 10.16 cm ion collector plates with a shorter guard plate at the rear to prevent field fringing in the ion collection region. The chamber which is used at wavelengths shorter than 58 nm has the anode extended to very near the ion collectors to reduce the field acting on electrons resulting from ionization events [4]. Direct calibrations of photodiodes are possible from 5–92 nm.

### 2.4 Thermopile

A windowless thermopile (see section 4) is used to transfer the capability of absolute detection by the ionization chamber to longer wavelengths. (In the region > 100 nm the photon energy is insufficient to ionize any practical rare gas.) The thermopile employed is an extended junction, windowless device which uses very thin gold leaves coated with thin gold black to form a detecting area which is effectively much larger than the actual thermocouples. Such a detector is in no way absolute and must be calibrated by reference to an absolute detector, in our case, the rare gas ionization chamber. Detection of relatively weak UV energy with a thermal detector is extremely difficult, and to achieve reasonable signal-to-noise it is necessary to use ac phase-locked techniques. Further discussion of this application of a thermopile detector is given in section 4.

## 3. Calibration Techniques

Although the exact calibration procedures differ in various portions of the spectral region covered, in all cases the absolute reference standard is the rare gas ionization chamber [3,4]. At wavelengths at which the photon energy is sufficient to ionize a rare gas, calibration of a photodiode can be accomplished directly; otherwise a windowless thermopile is used to transfer the calibration in wavelength. The mathematical treatment of the absolute calibrations follows.

### 3.1 Calibration Principles

#### 3.1.1 Ionization Chamber (long wavelengths)

The use of the rare gas ionization chamber in the 50–92 nm region is fairly straightforward, since the light source and grating used in this region preclude the existence of second or higher orders from the grating, and the photon energies are insufficient to ionize the appropriate rare gas more than singly and the electrons resulting from ionization events have insufficient energy to cause secondary ionization of the gas. The so-called double ionization chamber is used exclusively in this region, obviating the need for pressure and temperature measurements, or knowledge of the cross section of the gas.

The theory is treated in reference [3]. The fundamental equation from which the radiant flux may be calculated is:

$$I=\frac{{i_{1}}^{2}}{e\left(i_{1}-i_{2}\right)}$$ (1)

where
  **example***i*_1_=ion current (plate 1)1×10^−10^ A (C/s)*i*_2_=ion current (plate 2)1×10^−11^ A*e* = the electronic charge1.6×10^−19^ C*I* = radiant flux entering chamber6.944×10^8^ photons/s

The numbers shown at the right are examples of a “typical” hypothetical case.

##### 3.1.1.1 Direct Calibration of Photodiodes

If a windowless photodiode is being calibrated directly by use of the ionization chamber, the relationship

$$E=\frac{i_{D}}{e\;I\;k}$$ (2)

where

*i*_D_: = the emissive photocurrent from the photodiode
*I*: = the radiant flux incident on the photocathode
*E*: = the quantum efficiency (electrons/photon) of the photodiode
*k*: = the correction for monochromator gas absorption (in the ionization chamber mode)

may be used to determine the quantum efficiency. Combining eqs (1) and (2) gives:

$$E=\frac{i_{D}\left(i_{1}-i_{2}\right)}{{i_{1}}^{2}\;k}.$$ (3)

(In principle, if the currents from both the ionization chamber plates and the photodiode are measured with the same picoammeter using the same feedback resistors, absolute calibration of the picoammeter is not required.)

##### 3.1.1.2 Calibration of Thermopile

There is no need to obtain an absolute thermopile calibration if the response of thermopile to incident radiant flux can be determined relative to a stable reference. Such a reference, a mercury battery, is used to do this, enabling easy relative calibration of the ac thermopile system with the dc ionization chamber.

The general relationship describing the calibration of the thermopile, which is easily derived is:

$$I=\frac{{i_{1}}^{2}}{e\left(i_{1}-i_{2}\right)}=\frac{F\;R\;l\lambda }{k}\;\mathrm{so}\;F=\frac{k{i_{1}}^{2}}{R\;l\;\lambda \;e\;\left(i_{1}-i_{2}\right)}$$ (4)

where

*l*: = thermopile photoelectric correction
*I*: = radiant flux incident on thermopile or ion chamber (photons/s)
*F*: = thermopile calibration factor
λ: = wavelength (A) (to normalize irradiance)
*R*: = ratio of thermopile signal to test signal

The subsequent calibration of a photodiode using the thermopile is given by:

$$I\prime =\frac{i_{D}}{e\;E}=F\;R\prime l\lambda \;\mathrm{so}\;E=\frac{i_{D}}{e\;F\;R\prime l\;\lambda }.$$ (5)

(Here “*I*” and “*R*” have the same meaning as above, but are different values, since the thermopile and photodiode calibrations are done separately.)

#### 3.1.2 Ionization Chamber (short wavelengths)

The use of the rare gas ionization chamber in this region is complicated by the fact that photon energies are high enough to create both multiple ionization (more than one ion-electron pair per event) and secondary ionization from electrons with sufficient energy to ionize the gas. There is also an unrelated complication which the double ionization chamber helps to deal with: high order impurity in the diffracted light from the gratings used. Reference [10] describes a method for using the double ionization chamber to determine the fraction of the incident light which is first order. Since the range of photon energies used and the distribution of the continuum of SURF-II are now different from those described in the reference, the technique must be somewhat different.

In order to proceed, certain assumptions must be made. It is assumed that if there are high order impurities, they are only second order. It is also assumed that there exists a portion of the region in which there is only first order (appropriate choices of filters and machine energies can ensure this and the first assumption).

In regions where the fraction of first order is expected to be less than 1, determination of the incident radiant flux (normalized by use of a monitor) is made with both high and low pressure ion chamber measurements. The current from each of two equal length ion collector plates is measured independently in the high pressure mode (the ratio of currents will be seen to be needed); pressure and temperature data are measured in both modes. Two low pressure measurements are made at about 0.010 and 0.020 Torr presure and the normalization is extrapolated to zero pressure to eliminate any effects of secondary ionization (which cannot occur at zero pressure). The relationship which describes the amount of secondary ionization present during the high pressure measurement is then:

$$C=\frac{I_{H}}{I_{L}}$$ (6)

where

*C*: = secondary ionization coefficient
*I_L_*: = radiant flux entering the chamber (zero pressure)
*I_H_*: = radiant flux entering the chamber (high pressure)

In either pressure mode the flux is calculated from:

$$I=\frac{i}{e\left(1-\exp\left[-\mu \;kg\right]\right)}$$ (7)

where

*i*: = total ion current
*p*: = pressure
*T*: = temperature (C)
μ: = gas absorption coefficient (per cm)
*kg*: = $\frac{273.16}{\left(T+273.16\right)}\cdot \frac{p}{760}$.

To determine the fraction of first order “*f*” at a given wavelength, the above values of “*C*” are used with the data from the two ion collector plates (high pressure) in the relationship:

$$f=\frac{m_{2}\left(1-\tau b\right)}{\tau \left(m_{1}a-m_{2}b\right)+m_{2}-m_{1}}$$ (8)

where

*a*: = exponential term at *T, P* for primary wavelength
*b*: = exponential term at *T*, *P* for half wavelength
*c*_1_: = secondary ionization correction coefficient at primary wavelength
*c*_2_: = secondary ionization correction coefficient at half wavelength
*m*_1_: = *C*_1_(1*−a*)
*m*_2_: = *C*_2_(1*−b*)
τ: = $\frac{i_{1}}{i_{2}}$

Only low pressure ionization chamber data are used to determine photodiode quantum efficiencies in the 5–50 nm region. The equations describing the contributions to total ion current are:

$$\mathrm{ion}\;\text{current}\;\text{from}\;1\mathrm{st}\;\text{order}\;{\bar{i}}_{1}=\frac{fQ_{1}i}{Q_{2}K_{2}\left(1-f\right)+fQ_{1}K_{1}}$$ (9)

$$\mathrm{ion}\;\text{current}\;\text{for}\;2\mathrm{nd}\;\text{order}\;{\bar{i}}_{2}=\frac{i-{\bar{i}}_{1}K_{1}}{K_{2}}$$ (10)

where

*f*: = fraction of total flux which is first order
*Q*_1_: = absorption term for 1st order
*Q*_2_: = absorption term for 2nd order
*K*_1_, *K*_2_: = multiple ionization corrections for 1st and 2nd orders

The relationships for the general case in which the fraction of first order (*f*) is less than 1 are:

$$\text{Radiant}\;\text{flux}\;\text{at}\;\text{primary}\;\text{wavelength}:\;I_{1}=\frac{{\bar{i}}_{1}}{e\;Q_{1}}$$ (11)

$$\text{Radiant}\;\text{flux}\;\text{at}\;\text{half}\;\text{wavelength}:\;I_{2}=\frac{{\bar{i}}_{2}}{e\;Q_{2}}$$ (12)

The above calculations are used, with a prior measurement of the quantum efficiency at the half wavelength (where *f* = 1) to arrive at the efficiency at the primary wavelength:

$$QE\;\text{at}\;\text{primary}\;\text{wavelength}\;E_{1}=\frac{i_{D}-E_{2}\;e\;I_{2}}{e\;I_{1}}$$ (13)

where

*i*_D_: = measured photodiode current
*E*_2_: = *QE* at half wavelength
*I*_1_: = radiant flux at primary wavelength
*I*_2_: = radiant flux at half wavelength

### 3.2 Measurement Methods

#### 3.2.1 5–50 nm Region

Calibrations in this region are done at the NBS SURF-II facility, which is described in detail in section 4.1. The basic technique is to use the photoionization of a rare gas to calibrate a photodiode which can intercept the incoming light before it passes through a thin foil (preventing the ion chamber gas from flowing into the storage ring) into the ionization chamber. The calibration of the monitor photodiode is transferred to a working standard which is then used to calibrate outgoing photodiodes by intercomparison.

The calibration of the monitor photodiode is in terms of response per unit flux passing through the ionization chamber foil (as a function of wavelength). (It is, of course, impossible to operate a photodiode in the presence of ionization chamber gas.) The calibration of the monitor photodiode must be transferred to a working standard which is illuminated through the same foil(s) that were used during ionization chamber measurements.

#### 3.2.2 50–122 nm Region

In this region calibrations are done using a measurement system incorporating a normal incidence monochromator and a duoplasmatron light source. This system is described in detail in section 4.3. Absolute calibrations of a working standard windowless photodiode are done by direct interchange with a rare gas ionization chamber in the region 50–92 nm. Two additional wavelengths, 102.6 nm and 121.6 nm cannot be done using the ionization chamber directly, so the thermopile technique described in the next section is applied directly to a working standard at 102.6 nm, and the 121.6 nm point is obtained from intercomparison with a windowed standard. It is not necessary to correct for any of the sources of spurious ionization chamber data described in section 3.1 since the photon energy is always less than twice the ionization potential and there is no possibility of second order contamination in the exit beam (due to the use of a line source and a monochromator grating with very low efficiency at wavelengths below 50 nm). After a working standard has been calibrated, it may be used to calibrate outgoing photodiodes by direct intercomparison.

#### 3.2.3 116–254 nm Region

Calibrations in this region require the use of a thermopile to transfer the calibration from the ionization chamber (which cannot be used at wavelengths longer than 102 nm) to windowed photodiodes (which cannot be used at wavelengths shorter than 113 nm). Early studies proved that the appropriate thermopile appears to have the same sensitivity (probable error 3%) throughout the spectral region 58–92 nm [5,6]. Therefore it is proper to calibrate such a thermopile at ionization chamber wavelengths (several should give equal sensitivity), and then use the calibrated thermopile to calibrate a photodiode at longer wavelengths. This basic procedure is described in detail in section 4.2.

As an additional check on the thermopile-derived results, a low pressure Hg arc filtered lamp is used to determine the quantum efficiency of photodiodes. This lamp was calibrated by the Radiometric Physics Division of NBS by techniques traceable to blackbody radiometry, and is used as a single wavelength (253.7 nm) irradiance source, with the irradiance specified at 2 m distance from the source. Two such calibrated lamps are available

Outgoing calibrations of photodiodes are accomplished by intercomparison of known and unknown, measuring the ratio of photocurrents in a constant intensity monochromatic beam from the monochromator mentioned in section 4.2.

## 4. Calibration Systems

### 4.1 SURF-II Detector Calibration System (5–50 nm)

#### 4.1.1 History

The capability of calibrating transfer standard photodiodes at wavelengths requiring grazing incidence optics was established at NBS in the mid-1970’s making use of the SURF-II electron storage ring synchrotron radiation facility. This facility served as an extension to shorter wavelengths of the program which was already in place, in which both windowless and windowed transfer standards were made available to interested users. In both facilities the absolute reference detector was a rare gas ionization chamber.

Initially the SURF-II facility was used at wavelengths from about 20–50 nm [6]; eventually the range was extended to 5 nm. The transfer standards that were calibrated and issued were exclusively of the windowless NBS design, but on occasions the SURF-II facility was used to calibrate other special detectors for special needs of users [8,9].

In 1983 the existing facility was dismantled to make way for an incoming experiment, and the construction of a new apparatus was begun. A new, dual toroidal grating monochromator optimized for the 3–60 nm region was designed and constructed, as were the toroidal gratings for it. Also designed was a new experimental system incorporating the experiences of the original SURF-II detector facility. The new system was to provide greater flux levels at the experiment, coverage to shorter wavelengths, more accurate results, and faster throughput of calibrations.

#### 4.1.2 Experiment

**Figure 5:**
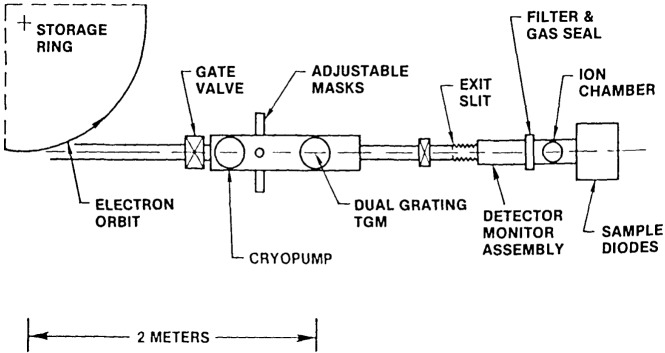
Schematic of Far Ultraviolet Detector Calibration Facility at SURF-II.

The basic configuration of the experimental system is shown schematically in figure 5. A pneumatic gate valve isolates the calibration facility from the storage ring. The central portion of this valve is fitted with a window to allow transmission of visible light in the closed position as an aid in system alignment. A pneumatically actuated shutter intercepts the incoming beam, and manually adjustable vertical and horizontal masks limit the illumination of the monochromator grating as appropriate.

##### 4.1.2.1 Monochromator

A sine bar drive monochromator with two interchangeable toroidal gratings is used. Dispersion is in the vertical plane, with the center 2 meters from the electron orbit tangent point. The angle of incidence at zero order is 83.5 degrees. The ruled gold gratings may be interchanged manually from outside the vacuum housing. At the shortest wavelengths (3–20 nm) a 1200 line/mm grating is used; a 300 line/mm grating is used at longer wavelengths. The spectral scanning range of the monochromator is from slightly below 0 to 20 nm (1200/mm grating) and ultimate resolution is .02 nm. The image of the electron beam is focused on an axially adjustable exit slit, with interchangeable prealigned slits available. A small pneumatic gate valve isolates the monochromator from the experimental chamber portion of the system.

##### 4.1.2.2 Foil Chamber

A bellows couples the monochromator assembly to the balance of the system, with a 4 mm diameter capillary limiting gas flow toward the monochromator. Radiation emerging from the capillary may be intercepted by a windowless monitor photodiode (of the same type that is ordinarily calibrated) with interchangeable polypropylene or aluminum filters. This assembly (not shown in fig. 5) provides the intensity level reference for all calibration activities via photocurrent from the photodiode.

##### 4.1.2.3 Ion Chamber

When the monitor detector and filter are not in the beam, radiation falls into the rare gas ion chamber through one of two manually interchangeable filters (as above) which also serve as gas seals during ion chamber activities, when the gas pressure may be as high as 2 Torr. These filters may be interchanged from outside the vacuum system. When non-ion chamber measurements are in progress, the chamber plates remain in place and have, in the absence of interacting rare gas molecules, no effect on the radiation beam.

##### 4.1.2.4 Intercomparison Module

At the rear of the apparatus is an easily removable flange containing an internal mounting wheel for six photodiodes which may be used to make intercomparison measurements. Electrical contact to the photocathode in the beam is made by an external pneumatic actuator, allowing photocurrent from this detector to be measured.

##### 4.1.2.5 Data Acquisition

A Digital Equipment LSI-11/23 computer and CAMAC interface modules accomplish most data acquisition activities remotely from the SURF-II control room. Stepping motors control both the wavelength drive and diode intercomparison wheel rotation, with encoders monitoring shaft positions. Pneumatic actuators control the shutter, monitor detector and filter positions, and photocathode contact at the intercomparison wheel. Status switches monitor the positions of shutter, monitor detector and filter, gas seal filters, and photocathode actuator. The SURF beam current monitor line is connected to enable integration periods to be normalized regardless of beam intensity level. Incoming data from either emissive photocurrent or ion current are converted to pulses whose frequency is proportional to the current intensity. The pulses are then counted for the period determined by reference to the house beam monitor. Three electrometers are used, with each calibrated daily using a standard current source.

The concept of system operation is that the monitor diode package (diode + filter) monitors the beam intensity and provides a reference for all ion chamber measurements. In other words, the package response is related to the radiation passing through the ion chamber filter. However, when the calibration of the package is transferred to a diode on the rear wheel, the same filter is in the beam, so it is thus possible to have knowledge of the magnitude of radiation reaching the rear detector, and to arrive at an absolute calibration of the detector. (The calibration of the monitor package is, of course, only relative.)

##### 4.1.2.6 Vacuum Systems

**Figure 6:**
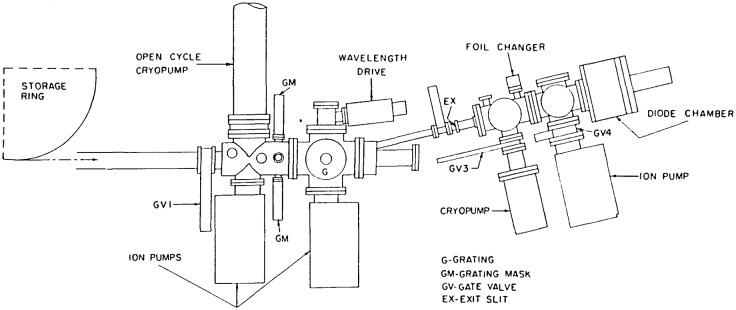
Detailed configuration of Far Ultraviolet Detector Facility at SURF-II.

The vacuum components associated with the SURF-II detector calibration system are shown schematically in figure 6. Pumping in the monochromator section of the apparatus is accomplished by two 110 1/s triode ion pumps, with an open cycle liquid helium cryopump over a gate valve available if needed. A nude ion gauge monitors pressure and a residual gas analyzer head is resident for SURF-II beam line acceptance studies. Beneath the foil chamber is a closed cycle cryopump with an isolation valve and ion gauge. The diode intercomparison/ion chamber region is pumped by another 110 1/s triode ion pump. Both this region and the foil chamber have ion gauges. A dedicated roughing stand consisting of carbon vane mechanical and liquid nitrogen sorption pumps is connected to the system through a valve, as is the rare gas supply system.

### 4.2 Thermopile System (100–320 nm)

#### 4.2.1 Introduction

At wavelengths below 102 nm, a rare gas double ionization chamber, which will count individual absorbed photons by the ion-electron pair produced in each absorption, can be used as an absolute photon detector [3,4]. Working standard photodiodes in this wavelength range can therefore be calibrated using this ionization chamber (although they will not operate properly in the gas environment of the ionization chamber).

At wavelengths above 102 nm, the photon energy is not sufficient to ionize xenon, the rare gas with the lowest ionization potential (12.13 eV). In this case a method must be found for transferring the absolute calibration of the ionization chamber to the longer wavelength range. Thermal detectors measure the heating of an absorbing element in proportion to the power of the incident radiation and have a response that is generally independent of wavelength. Thus a thermal detector can be calibrated against an ionization chamber in the short wavelength range and then used in turn to calibrate a working standard photodiode in the long wavelength range.

#### 4.2.2 Sources of Error

Thermopiles in particular have been studied as potential standards in the 100–300 nm range [5,6]. The assumption that a thermopile will develop a given voltage for a specific level of incident radiation, independent of wavelength, was tested with regard to four possible sources of error:

1. The thermopile may not absorb the same percentage of incident photons independent of wavelength.
2. The thermopile may have energy carried away by photoejected electrons.
3. The thermopile may have a wavelength-dependent time constant when ac response to chopped radiation is measured. This effect could cause wavelength-dependent variations in ac sensitivity.
4. The thermopile may have a wavelength-dependent spatial sensitivity.

Possibility 1) was checked for two typical gold blacks used on a windowless thermopile. The reflective scattering at three wavelengths in the vacuum UV was compared to that in the visible and near infrared and found to be the same within 1% of the incident intensity.

**Figure 7:**
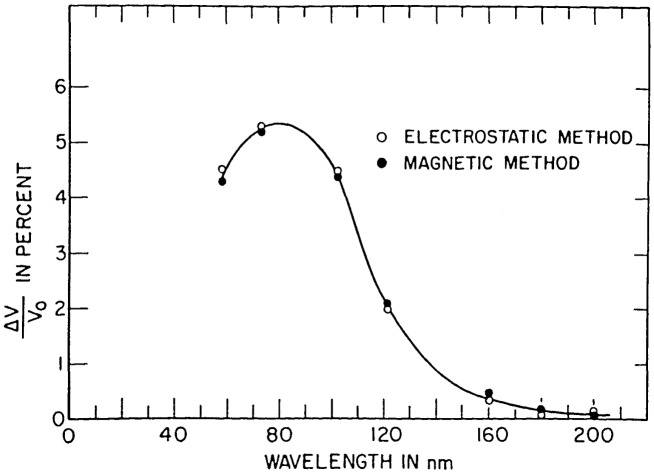
Wavelength dependence of thermopile signal loss due to photoejected electrons.

Possibility 2) was tested by measuring the thermopile signal with an electric or magnetic field either switched on to return photoejected electrons to the thermopile or switched off to let them escape. A correction curve (fig. 7) for the region 58–170 nm was derived to take account of this loss mechanism.

Possibilities 3) and 4) were tested by scanning a thermopile through a narrow beam in the far UV and visible with both dc and ac techniques. Both possibilities were found to have a negligible effect on the results.

The issue of whether such a thermopile indeed had wavelength-independent sensitivity was investigated by calibrating one both in the far UV (with a rare gas ionization chamber as an absolute detector), and in the visible/near infrared (using an NBS carbon filament lamp calibrated for total irradiance). The comparison agreed to about 3%, using the appropriate photoelectric corrections for the far UV data.

#### 4.2.3 Calibration Technique

After the demonstration that the thermopile is a reliable detector at longer wavelengths when calibrated by an ion chamber below 100 nm, a UV spectrometer and detector chamber were built to perform UV calibrations on working standard diodes from 100–300 nm [10]. The detection system used ac signal detection and processing, since the magnitude of the thermopile emf in the far UV is very weak compared to changes in the background blackbody radiation, which is also detected by a thermal detector in a dc mode.

Vacuum UV light is obtained from a 1-m normal incidence grating monochromator, equipped with a duoplasmatron light source. A 13 Hz chopper, located between the light source and the entrance slit, is used during thermopile measurements to interrupt the light entering the monochromator. Attached to the exit arm of the monochromator is a vacuum chamber which houses the ion chamber and thermopile.

The thermopile used is of the extended junction variety, employing three such junctions to give a sensitive area 1 mm×6 mm. It is essentially identical to the one used in the earlier study of thermopiles mentioned above. A rectangular mask with an opening slightly smaller than the area of the thermopile is used. A three point mounting, designed kinematically for accurate relocation, positions the thermopile relative to the surface of the mask, about 0.5 mm from its surface. The thermopile signal is amplified by a 13-Hz amplifier, rectified synchronously with the chopping frequency and recorded using calibrated picoammeters. Net emf readings are taken and referenced to a stable ac test voltage that is applied across the thermopile in the absence of radiation. Immediately following thermopile data acquisition, the thermopile is rotated away from the mask, and the ion chamber or diode being calibrated is exposed to the monochromatized light. The ion chamber or diode currents are then measured. (A gas manifold and set of pneumatic valves are programmed to initiate the appropriate flow of gas before the ion chamber current measurements are made. After the measurements the gas is pumped away.) The thermopile is then rotated back onto its kinematic mounts behind the mask for another measurement. Alternating measurements are made in this way until enough data have been accumulated to overcome the signal-to-noise problems of the thermopile in the far UV.

An additional, independent calibration of the standard diode is made at 253.7 nm. At this wavelength a stabilized low pressure Hg lamp, calibrated for spectral irradiance at this wavelength by blackbody radiometry techniques, is used as a spectral irradiance standard. The diode quantum efficiency is determined from the known flux falling on the diode and the measured diode current.

#### 4.2.4 System Description

**Figure 8:**
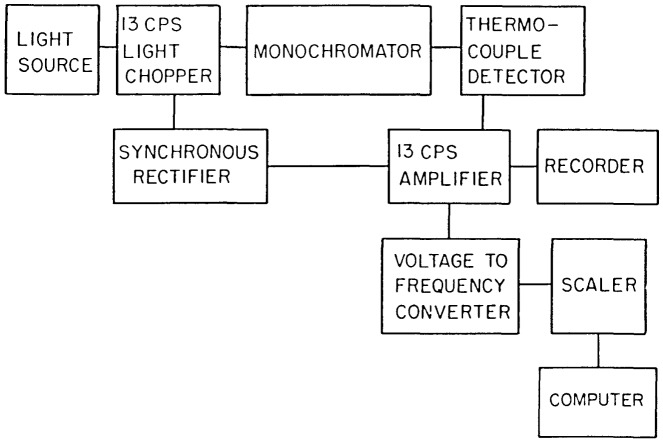
Schematic of thermopile detector system.

The system as it is presently operated is shown schematically in figure 8, and consists of three distinct parts:

1. Duoplasmatron light source
2. Grating Monochromator
3. Detector chamber

**Figure 9:**
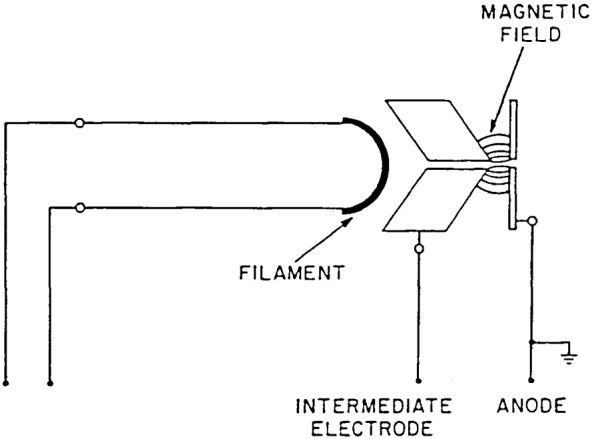
Duoplasmatron light source.

The duoplasmatron light source is a three electrode device, composed of a Pt mesh filament coated with an emitting material, a baffle electrode, and an anode (see fig. 9). A low pressure arc discharge is constricted by the funnel-shaped baffle and the anode. An axial magnetic field produced by a cylindrical permanent magnet surrounding the outer shell of the light source further constricts the discharge to a narrow plasma beam along the axis. Helium is used for 58.4 nm, neon for 73.6 nm, argon for 92.0 nm, and hydrogen for the 116–320 nm region. The first three gases are used in the ion chamber-thermopile calibration, and hydrogen for the standard diode vs. thermopile calibration. The major arc currents are normally 1.5 A for the rare gases, and 1.1 A for hydrogen.

A chopper blade is located between the light source and the monochromator entrance slit, and is driven in a reciprocating motion through a bellows by a small motor immediately below the light source housing. A flap valve, operated by a stepping motor, is placed after the chopper blade and can be rotated either to seal off the light source from the monochromator, to block the light but allow pumping of the light source through the entrance slit, or to let the light enter the monochromator. The entrance slit is vertical and has a micrometer adjustment of 200 microns per turn.

The grating monochromator is a 1-m normal incidence monochromator with a spherical grating of 600 lines/mm and a dispersion at the exit slit of 1.67 nm/mm. The dispersion plane is horizontal. The monochromator is pumped by 6″ oil diffusion pump with a freon-refrigerated baffle to reduce oil contamination in the monochromator chamber. A pneumatic gate valve above the freon baffle is electrically connected to a presure-sensing relay, and closes automatically if the system pressure rises above a preset value. This arrangement protects the system from unforeseen pressure bursts or leaks. A separate roughing valve allows the monochromator to be pumped down from atmospheric pressure without turning off the diffusion pump.

The detector chamber contains the thermopile in its rotatable mount, and has provision for placing the double ionization chamber or the diode to be calibrated in the light beam passing through the exit slit (see fig. 4). The exit slit is a fixed vertical slit 0.8 mm wide, corresponding to about 1.3 nm band width.

**Figure 10:**
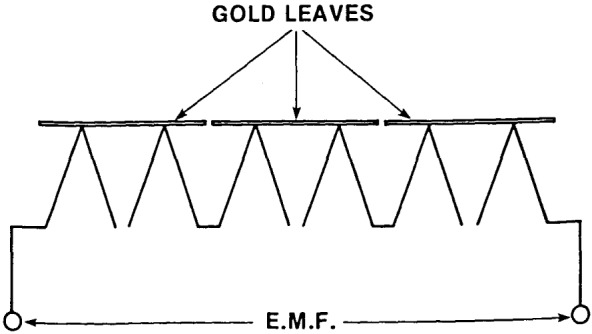
Schematic of thermopile detector.

The thermopile (fig. 10) is a series connection of three elements which are composed of bismuth-tellurium and bismuth-antimony alloys. The thermal junctions are extended using gold flakes of 1 mm×2 mm area. Together they form a sensitive area 1 mm×6 mm. The thermopile is compensated, designed for a 13 Hz chopping frequency and constructed entirely of nonmagnetic materials. The time constant of the thermopile is about 0.04 s, and has a typical sensitivity (in vacuum) of 1 microvolt/microwatt and an ENI (equivalent noise input) power of approximately 10^−9^ W. An impedance-matching transformer is used between the thermopile and a preamplifier.

The signal from the preamplifier is fed to a 13 Hz broadly tuned amplifier. The output of this amplifier is rectified synchronously with the chopping frequency by a mechanically coupled rectifier. The signal is then filtered, further amplified, and fed into a V-F converter. A small computer is used to record the data and perform statistical evaluation of the results.

A flap valve is mounted 6 cm in front of the mask, and when closed allows the detector chamber to be let up to atmospheric pressure without affecting the rest of the system. The rear section of the detector chamber is bolted to the rest of the chamber using a Viton gasket as a vacuum seal. Entrance to the detector chamber is obtained by removing this rear section.

The thermopile is attached to a rotatable mounting so that, under computer control, it may be brought into registration with the kinematic mount, or rotated between the plates of the ion chamber out of the beam.

The ion chamber used is a double ion chamber similar to that described by Samson [3] (see also section 2). The length of each collector plate is 10.2 cm, and the forward end of the first plate is positioned in the plane of the mask. The axis of the light beam is 0.8 cm from the positive plate, which, with the mask, is kept at +22 V with respect to the collector plates. The ion chamber assembly is a self-contained unit with teflon bars used as framework to support the various polished stainless steel plates. The assembly is positioned behind the mask reproducibly by a locating screw and machined recess in the floor of the chamber.

Two corrections applied to the data as they are recorded (i.e. in real time) are as follows:

1. The thermopile signal is corrected for losses through photoemission, amounting to 4.4% at 58.4 nm, 5.3% at 73.6 nm, and 5.1% at 92.0 nm (fig. 7).
2. The reduction in flux entering the ion chamber due to absorption by the argon or xenon leaking into the monochromator through the exit slit is corrected for by making a separate measurement of attenuation vs. monochromator pressure over a range of pressures. Thus by monitoring the monochromator pressure the appropriate correction, typically 4%, can be applied.

The MgF_2_-windowed diodes are then calibrated against the thermopile at 23 wavelengths from 116.4 to 253.7 nm. The light source gas is hydrogen over this entire wavelength range. From 116.4 to 160.8 nm, the hydrogen line spectrum is used. Above 160.8 nm the emission is from the molecular hydrogen continuum. A quartz filter is placed in front of the light source when wavelengths longer than 160.8 nm are used to prevent second order radiation from reaching the thermopile and diode.

### 4.3 Photodiode Intercomparison System (50–254 nm)

#### 4.3.1 History

In 1968 a system was built whose primary purpose was to calibrate outgoing transfer standard detectors by intercomparison with NBS working standards which had been calibrated by the already existing thermopile system [5]. Initially, this system lacked any control or data reduction capabilities; this was added in the early 1970’s and upgraded in an evolutionary fashion to the present configuration, in which nearly all data acquisition and reduction functions are automated. With this evolution came the additional capability of performing absolute calibrations of windowless photodiodes in the 50–92 nm spectral region, which greatly expanded the coverage possible and considerably reduced the time required. In the present form, intercomparison-type calibrations are conducted from 50 nm to 254 nm, and absolute calibrations from 50 nm to 92 nm.

#### 4.3.2 Experiment

The basic configuration of the experimental system is very much the same as that of the thermopile system. A duoplasmatron light source illuminates the entrance slit of a normal-incidence vacuum monochromator, and the experimental chamber is attached to the exit slit flange. Various experiments are permanently attached to modular flanges, which can be exchanged on the otherwise empty chamber. Data acquisition and reduction is under the control of a programmable calculator.

##### 4.3.2.1 Monochromator

A one meter normal-incidence McPherson model 225 monochromator is used with its own vacuum pumping system. A 15 cfm mechanical forepump is used for both rough pumping and for backing a 7″ diffusion pump, which uses DC705 silicone pumping fluid. A refrigerated chevron trap and pneumatic gate valve are attached to the top of the pump. A zeolite molecular sieve trap is used in the foreline to minimize the migration of forepump oil vapors into the high vacuum system. Both exit and entrance slits are laterally adjustable, and both the exit and entrance flanges may be isolated from the monochromator vacuum by manual flap valves. The diffraction grating is a 600/mm replica, with nominal blaze at 150 nm. The dispersion at the exit slit is thus 1.67 nm/mm. A modified sine-bar drive provides wavelength scanning by both rotation and translation of the grating.

##### 4.3.2.2 Light Source

A duoplasmatron source, fabricated in-house, is used for all spectral regions in this system. A manifold allows the introduction of any of several gases as appropriate for the emission desired, and a roughing line is attached to the foreline of the experimental system. This type of source uses a hot filament, three electrodes and a pinching magnetic field to create a rather dense plasma which is on the optic axis of the monochromator. Because it is only about 1.5 mm in diameter and is several cm from the entrance slit, only the central portion of the grating is illuminated by the plasma. Gases routinely used at wavelengths short of 100 nm are helium, argon, krypton, and neon. For wavelengths longer than 100 nm hydrogen is used. Cooling of the source is by forced air both in the area where the plasma strikes the anode and around the exterior of the filament end. Separate unregulated power supplies are used for the filament (ac) and the minor and major arcs (dc). A cross section of the duoplasmatron is shown in figure 9.

##### 4.3.2.3 Experimental Chamber

A cylindrical stainless steel chamber with provision for rear mounting of modular flanges is attached to the exit slit flange. This chamber is provided with an independent vacuum system, consisting of a 2″ pneumatic gate valve over a refrigerated chevron trap over a 2*″* diffusion pump using polyphenyl ether fluid. The 2″ pump is backed with a trapped mechanical forepump, which is also used for roughing. The usual valves isolate and protect. Experiments are attached to the chamber mounted on a flange, which attaches to the rear of the chamber.

##### 4.3.2.4 Experimental Modules

Three such modules are routinely used: an intercomparison module, a mapping module, and an ion chamber module.

The intercomparison module contains a rotating wheel, on which two photodiodes may be mounted for intercomparison measurements. In general, the photocathodes of the two photodiodes are shorted to ground, the exception being that when either is in the beam, its photocathode is connected by means of a gold wiper to a vacuum feedthrough. The anodes are tied together and at all times connected to another feedthrough. Provision is made for rotational relocation of either photodiode about its center, and quartz or Vycor filters may be manually inserted in the beam to aid in eliminating overlapping orders orders from the grating.

The mapping module flange has orthogonal linear motions on the atmospheric side driving a shaft going through the flange with a bellows seal. The shaft is supported inside the vacuum by a gimbal mount, so that displacement of the outside end of the shaft will result in a proportional displacement of the end inside the vacuum. Thus an object may be scanned in space inside the vacuum system in a controlled manner. The normal applications of this module would be either to map the response of a photocathode (with the beam stopped by a fixed small aperture) or to map the intensity distribution of the beam (with the small aperture attached to a photodiode). The external motions can be driven by stepping motors, and the exact position may be monitored by linear displacement transducers.

The ion chamber module is used to directly calibrate a windowless photodiode by means of a rare gas double ion chamber [3,4]. Attached to a rotary motion on the vacuum side of the flange are an ion chamber with two 10 cm plates with a guard plate and a photodiode. Either one or the other may be placed in the beam by a stepping motor. As this module is placed into the experimental chamber a small teflon flange near the exit slit provides a seal so that the only leakage path for gas from the chamber into the monochromator is through a small aperture in the exit beam. High purity argon and xenon are individually brought into the chamber on demand with needle valves limiting the flow so as to balance the influx and leakage into the monochromator and give the desired gas pressure. The gate valve beneath the chamber may be program controlled to open and remove the residual rare gas before photodiode readings are taken and to close so that fresh gas may be admitted before making ion current readings.

##### 4.3.2.5 Data Acquisition

Almost all system operations are controlled by a programmable calculator with an external CPU and interfaces to drive stepping motors, actuate solid state relays and hence valves, etc., switch between sources of very low current or analog voltages and actuate and read a counter-timer. Analog voltages are proportionally converted to pulses, which are counted for fixed periods by the counter-timer. The control programs also contain the data reduction routines, so that the final results of an experiment are available at the conclusion of the data acquisition sequences.

## 5. Treatment of Errors

Uncertainties associated with the measurement of quantum efficiencies are given in each Report of Test as “probable errors,” meaning that there is a probability of 0.5 that the true quantum efficiency values lie within the stated error range about the values quoted. The errors represent the expected accuracy of the sample photodiode when properly used in a customer’s system, without regard for any systematic errors present in that system.

The probable error values are determined by summation, in quadrature, of estimates of the effect of all sources of systematic errors in the NBS measurements, including the precision of repeated measurements, on the determination of the quantum efficiency of a photodiode. The analysis of the calibration of windowed photodiodes in the thermopile system follows.

The sources of possible error are shown in table 1. Those sources associated with current measurements are due primarily to the probable error of the current source used to calibrate the measuring picoammeters. The gas absorption uncertainty involves the repeatability of ion gauge measurements (the calibration of such gauges has been observed to drift in time). The photoelectric correction is an empirically determined quantity (see section 4.2) which may vary from the samples originally measured.

The cathode uniformity is measured on each photodiode and units with more than 5% variation in pixel spatial response over the central 1 cm diameter area are rejected. (Should a photodiode be found to have a discontinuous uniformity function, even within the 5% acceptance criterion, it would not be issued as a transfer standard.) The portion of the measured area actually used in the NBS measurements is the central 4×5 mm section, and the intensity distribution in the incident beam is relatively uniform over much of this area. The ultimate user of a photodiode will also illuminate the central area of the photocathode, but with a generally different intensity distribution and, perhaps, a somewhat larger area illuminated. This would result in an error in the transfer of the NBS calibration, but not approaching the peak variation of 5% allowed in device acceptance. Our estimate is a probable error of 2% from this source.

The variation of thermopile sensitivity (with wavelength) is an estimate based on the variation seen within the 58–92 nm range and the cross-calibration of detectors using a calibrated mercury source (section 3). The measurement precision (repeatability) is a function of wavelength in the above case in the upper portion of the windowed photodiode calibration range because of deteriorating signal-to-noise in the thermopile measurements as the efficiency of the optics declines and the intensity of the beam is thus reduced.

The above example covers the case of uncertainties in the calibration of windowed photodiodes. The probable error currently being quoted for windowless photodiode calibrations is 10–15% in the 5–50 nm region and 8% in the 50–122 nm region. These errors are determined from the analyses displayed in tables 2 and 3.

In the table 2 analysis, the cross section and multiple yield data are taken from published sources with interpolation between published wavelengths where necessary. Since, in this region, the reduction of ionization chamber data entails the use of gas constants, including the pressure and temperature, the contribution to the error budget from these must be included. Errors arising from measurements of current are primarily from the variance within sets of three consecutive conversions of currents permitted in the acquisition program. The radiation impurity contribution can only be estimated from observation of system performance, and could include improper assessment of overlapping orders from the diffraction gratings, stray light from masks, baffles, walls, etc., the “zero order tail” from the gratings, and imperfections in the filters used to minimize this source of error. This error would be wavelength dependent, of course.

All current measurements in the region covered in table 2 are conducted with a single picoammeter, and since it is the ratio of photodiode to ion chamber currents that is used to calculate quantum efficiency, an absolute calibration of this picoammeter is not necessary. The estimated uncertainties associated with current measurements would be largely due to ionization of residual gases and other spurious effects. The gas absorption correction is necessary only during ion chamber measurements to account for the radiant flux absorbed in the monochromator (typically 10% or less) so the likely error from this source is minimal. The stability of the quantum efficiency of working standard photodiodes is felt to be a possible source of error only in the longer wavelength region (50–122 nm), since in the 5–50 nm region (at SURF-II) the photodiodes are calibrated and stored in ultrahigh vacuum, whereas in the other facility calibrations are in an oil-pumped system, and storage is in air.

## Figures and Tables

**Table 1:** Thermopile-based calibrations.

Error Source	Estimated Uncertainty (%)
**Calibration of thermopile (58–92 nm):**	
ion current (electrometer manufacturer)	2
gas absorption (estimated)	1
photoelectric correction (estimated)	0–1
measurement precision (observed)	3
radiation impurity (estimated)	0–0.5
**Photodiode calibration by thermopile (116–253 nm):**	
photocurrent (electrometer manufacturer)	2
photoelectric correction (estimated)	0–1
cathode uniformity (observed)	2
thermopile wavelength sensitivity variation (estimated)	3
measurement precision (observed)	2–8
radiation impurity (estimated)	0–1
**Probable Error (%)**	**6–10**

**Table 2:** Windowless photodiode calibrations at SURF (5–50 nm).

Error Source	Estimated Uncertainty (%)
gas cross sections (data reference)	3–4
multiple yield (data reference)	0–1
gas temperature (estimated)	0–0.5
gas pressure (transducer manufacturer	0–3
photocurrent precision (observed)	2–6
ion current precision (observed)	5–7
radiation impurity (estimated)	8–12
**Probable Error (%)**	**10–15**

**Table 3:** Windowless photodiode calibrations (non-SURF, 50–122 nm).

Error Source	Estimated Uncertainty (%)
photodiode current (estimated)	2
ion current (estimated)	2
gas absorption correction (estimated)	0–1
measurement precision (observed)	3
stability of quantum efficiency (estimated)	7
radiation impurity (estimated)	0–1
**Probable Error (%)**	**8**

## Footnotes

1 Figures in brackets indicate literature references.

2 Commercial products–materials and instruments–are identified in this document for the sole purpose of adequately describing experimental or test procedures. In no event does such identification imply recommendation or endorsement by the National Bureau of Standards of a particular product; nor does it imply that a named material or instrument is necessarily the best available for the purpose it serves.
